# The arrow of time: Advancing insights into action control from the arrow version of the Eriksen flanker task

**DOI:** 10.3758/s13414-020-02167-z

**Published:** 2020-10-25

**Authors:** K. Richard Ridderinkhof, Scott A. Wylie, Wery P. M. van den Wildenberg, Theodore R. Bashore, Maurits W. van der Molen

**Affiliations:** 1 Department of Psychology, University of Amsterdam, Amsterdam, the Netherlands; 2 Neurological Surgery, University of Louisville School of Medicine, Louisville, KY USA; 3 Psychology, University of Northern Colorado, Greeley, CO USA

**Keywords:** Cognitive control and automaticity, Inhibition, Perception and action

## Abstract

Since its introduction by B. A. Eriksen and C. W. Eriksen (*Perception & Psychophysics, 16*, 143–49, 1974), the flanker task has emerged as one of the most important experimental tasks in the history of cognitive psychology. The impact of a seemingly simple task design involving a target stimulus flanked on each side by a few task-irrelevant stimuli is astounding. It has inspired research across the fields of cognitive neuroscience, psychophysiology, neurology, psychiatry, and sports science. In our tribute to Charles W. (“Erik”) Eriksen, we (1) review the seminal papers originating from his lab in the 1970s that launched the paradigmatic task and laid the foundation for studies of action control, (2) describe the inception of the arrow version of the Eriksen flanker task, (3) articulate the conceptual and neural models of action control that emerged from studies of the arrows flanker task, and (4) illustrate the influential role of the arrows flanker task in disclosing developmental trends in action control, fundamental deficits in action control due to neuropsychiatric disorders, and enhanced action control among elite athletes.

A remarkably active and vibrant area of investigation in cognitive science is devoted to articulating the neurocognitive mechanisms that mediate action control. Action control refers to a subset of adaptive cognitive control processes involved in the coordination of one’s instantaneous urges vis-à-vis actions that concord with our intentions or instructions. Our responsiveness to action affordances (alluring and potentiating opportunities for action in a particular situation, some more potent than others; J. J. Gibson, 1979) is guided by our current concerns and intentions; we are responsive not to just *any* affordance, but to task-*relevant* affordances. Goals, concerns, prior experience, and instructions have shaped our sensitivity to relevant affordances, such that we are not always captivated by the one action affordance that happens to present the most potent solicitation. But what happens when we are captured by unsolicited action affordances? What mechanisms do our brains engage to control conflicting actions and mitigate interference with goal-directed behavior?

The literature on action control that addresses these fundamental questions encompasses many contributions that are based on the Eriksen flanker task, described in more detail below. While the original version of the task consisted of arrays of letters (a central target letter flanked on each side by letters that were either congruent or incongruent to the target), later versions used other types of symbols, such as colors, geometrical shapes, or arrows. The arrows version in particular has been used widely, in a variety of contexts, ranging from fundamental behavioral and neuroscientific studies to developmental, clinical, and sports science. Our aim at present is to provide a (necessarily selective) review of the influential contributions driven by the arrows version of the Eriksen flanker task, from its inception in the late 1980s until today.

## The seeds of action control: Seminal work on the Eriksen flanker task

Of particular interest in the study of action control is the competing influences between stimuli containing information that designate the desired response and stimuli containing task-irrelevant information that, nonetheless, activates responses that overlap or conflict with the desired response. A widely used task to examine this conflict-inducing scenario is the so-called Eriksen flanker task.

### The foundational paper: B. A. Eriksen and Eriksen (1974)

The Eriksens’ seminal 1974 introduction of the letter flanker task (first-authored by Erik’s wife Barbara, and now cited close to 4,000 times) was conceived to address empirical and conceptual deficiencies in the visual search literature that, theretofore, had failed to resolve the nature of visual search, target identification, and the effects of noise elements on target search speed and accuracy. This work built on Erik’s prior studies (e.g., C. W. Eriksen & Hoffman, 1972) using circular letter displays and precued target locations that had yielded three major findings: “First, attentional selectivity is unable to eliminate completely the effects of extraneous stimuli. . . . Second, the spatial proximity of noise letters to the target has a nonlinear effect upon target RT. . . . Third, the effect of noise letters on target RT is predominantly on the response side as opposed to the processing side . . . if the noise letters require a response opposite or incompatible to that of the target letter, a large impairment in RT is obtained. This finding would tend to place the locus of the effect in terms of response competition” (B. A. Eriksen & C. W. Eriksen, 1974, pp. 143–144).

From this incisive reasoning emerged what in contemporary cognitive science and cognitive neuroscience is considered the classic, paradigmatic flanker task, the progenitor of multiple generations of research.

The basic task design involved flanking a target stimulus (letter) with other letters that varied in their physical similarity to the target or in their mapping to the identical or to the opposite response as the target. The capital letters *H, K, S,* and *C* were used both as targets and as flankers. Letters with angular (*N/W/Z*) and circular features (*G/J/Q*), respectively, served as structurally similar and dissimilar flanking noise (E. J. Gibson, 1969). The target letter always appeared at the same location (i.e., at visual fixation), either alone or flanked by three letters on both the left and the right side. Participants responded to a target letter by moving a small lever to the right or to the left based on a pre-determined mapping of letters to a specific response. Two of the target letters (e.g., *H, K*) were assigned to a leftward directional response, and the other two target letters (e.g., *S, C*) to a rightward response. Combinations of target and flanker letters provided the critical conditions for examining the effect of noise on target processing. A target letter (e.g., *H*) could be flanked by the same letter (*HHHHHHH*), or by a different letter assigned to the same response mapping (congruent; e.g., *KKKHKKK*) or to the opposite response mapping (incongruent; e.g., *SSSHSSS*).^1^ Thus, this initial study was designed to assess the effect of conflicting flanker information, both perceptual-related and response-related, on target processing.

While the study addressed several hypotheses about the nature of visual search, two outcomes in particular provided the foundational patterns that would spawn the conceptual framework for action control. First, a target letter flanked by the identical letter or by a different letter that was associated with the same response as the target produced similar reaction times and accuracy rates. Second, marked slowing of RT and reduced response accuracy were induced when a target letter was flanked by incongruent letters that signaled the opposite response. On the basis of these patterns, the Eriksens concluded that the effect of noise is neither a “distraction effect” nor a “primitive perceptual process” nor “a rudimentary noting of the presence or absence of items in the visual field.” These speculations could be discarded, they argued, by their finding that RT was not influenced differentially by the physical similarity or dissimilarity of the noise letters and target letter. Slowing was only evident when the noise letter signaled the opposite, not the same, response.

On the basis of this pattern of factor effects, the Eriksens concluded that the slowing in RT was due primarily to what they called *response competition*, the flanking distractors being processed to a sufficient depth to activate the alternative response, which in turn must be inhibited before the correct response can be executed. In thus concluding, they argued against the consensus view that search effects were produced at the stimulus level of processing by shared featural characteristics between the target and the distractors. The clarity of the Eriksens’ arguments for developing the flanker task, the precision of their experimental methods, the strong empirical support for their hypotheses, and the compelling interpretations they advanced for the effects of noise on the response-end of processing laid a well-girded empirical and conceptual foundation for the field of action control.

### The transitional paper: C. W. Eriksen and Schultz (1979)

The empirical and conceptual foundation laid by the Eriksens in their first paper was built on, both empirically and conceptually, in the second paper from their lab, C. W. Eriksen and Schultz (1979). In this paper, comprised of a series of three experiments, the findings from the first paper were replicated and extended. Moreover, they introduced a conceptual framework within which to interpret their findings, the continuous flow conception. This conception provided the transition to mental chronometry. Thus, the first paper was foundational and the second paper was transitional. It set the empirical/conceptual stage for a transition from understanding visual search to characterizing the basic structural and chronometric properties of the cognitive processes mediating speeded decision-making, prominently including response conflict.

Eriksen and Schultz argued against models that located noise effects at the early levels of perceptual processing. They noted, on the basis of the 1974 B. A. Eriksen and Eriksen paper, that “subjects cannot restrict their attention to process only a single letter, even when the location of this letter is clearly designated and known beforehand”; and second, “the noise letters are processed along with the target to the point of incipient response activation, [as] follows from the salient finding that response-incompatible noise letters produced considerably greater impairment in reaction time than did response-compatible or neutral letters” (p. 251). Further, Eriksen and Schultz argued that the pattern of response interference ruled out “visual search models employing discrete successive stages of the form: input → central decision process → response activation” because it showed “an appreciable component of noise interference at the response level” which “would not happen if responses were activated only after a decision had been made” (p. 251). They then provided a *précis* of their continuous flow conception that captured its essence: “information about stimuli accumulates gradually in the visual system, and as it accumulates, responses are concurrently primed or partially activated” (p. 252).

To test this notion of continuous flow, two experiments manipulated variables their model suggested could either increase or decrease the level of activation of responses induced by the incongruent flanking noise relative to activation of the target response: making the target (a) smaller or larger, (b) less bright or brighter, or (c) lower or higher in contrast than the incongruent noise. Replicating earlier findings, incongruent flanker noise slowed response speed substantially. Moreover, response speed was observed to slow the least when the target was larger than the incongruent flankers, and to slow the most when the target contrast was lower than the flanker contrast or the target size was smaller than the size of flankers, because (in the authors’ view) the slower processing time for the target allows the competing noise response to achieve a higher level of priming and thus produce greater interference to the target response. An additional experiment manipulated the relative onset times of the target vis-à-vis flankers to test more directly the assumption of differential rate of buildup of competing responses. The magnitude of the interference effect produced by incongruent noise was anticipated to be greatest when the target and incongruent noise appeared simultaneously and to decrease gradually as target onset preceded flanker onset. Conversely, when the flanking noise preceded the target, response speeds to target letters were expected to be less and less affected by the noise stimuli as flankers onset preceded target onset. These patterns were observed as predicted (note, the size of the interference effect was largest when the noise occurred either 100 ms before or at the same time as the target).

On the basis of these combined findings, Eriksen et al. argued that parallel processing of multiple stimulus elements can proceed to the point of incipient response activation, where response competition can be evoked. Eriksen et al. reasoned that these effects were produced by a continuous flow of concurrent accumulating information emanating from the target and flankers that continuously activated the response associated with each and, as a result, induced response competition between the two for attainment of their respective thresholds for response production. The demonstration that unintended responses could be activated by spatially distinct, task-irrelevant stimulus information and that the level of response activation could be modulated experimentally provided the impetus for investigations to identify underlying neural mechanisms associated with response competition.

### The transformational paper: Coles, Gratton, Bashore, C. W. Eriksen, and Donchin (1985)

Eriksen et al. then set out to augment the traditional tools of mental chronometry with measures of the latency of the P300 component of the event-related brain potential (ERP) and measures of the electromyogram (EMG). Cognitive psychophysiological measures like the P300 and EMG provide indices of covert processes, not accessible to behavioral measures, associated with the speed of stimulus-relevant decision-making, or stimulus evaluation (P300 latency), and the execution of overt responses (EMG activation) that instantiate those processes. Coles et al. (1985) reasoned that the degree to which multiple responses are activated concurrently, and to which their activation slows target recognition and compromises overt response execution, can be articulated, respectively, by associated changes in P300 latency and EMG activation.

Making a target more difficult to identify by locating it in a surround of distractors slows both P300 latency and RT, while execution of an incompatible response increases RT time while having little or no effect on P300 latency (Magliero, Bashore, Coles, & Donchin, 1984; McCarthy & Donchin, 1981). Coles et al. exploited this dissociative/associative capacity, refining it further by measuring variations in EMG and concomitant response-device activation, partitioning these variations into different types of partial to full activation as indices of differing degrees of incorrect and correct response activation, and then assessing associations and dissociations of factor effects on these different types of response system activation. This partitioning allowed Coles et al. to directly test the hypothesis that the degree to which incongruent flankers slow RT is related to the degree to which they induce response competition. As expected, partial activation of incorrect responses was observed to occur more often in the incongruent condition and varying degrees of partial activation were associated with varying degrees of response speed slowing. Moreover, P300 latency (taken to reflect stimulus evaluation time) increased systematically as incorrect response activation (i.e., on the side contralateral to the correct response) became more egregious (from EMG only to response device), providing compelling support for the important contribution of *response competition* to the interference effects of noise at both the level of target evaluation and overt response execution. Hence, revealed in these measures of partial response activation of the peripheral musculature and in P300 latency were, respectively, the simultaneous activation and subsequent inhibition of the competing incorrect response and the concurrent slowing of stimulus evaluation induced by incongruent flanking noise that constitute the conceptual core of the flanker effect.

We will return to the issue of continuous flow in a later section; but first, it is time to introduce the arrow version of the Eriksen flanker task and its role in advancing our understanding of adaptive action control.

## Inception of the arrows flanker task

In beginning of this paper, we mentioned a range of stimuli that had been substituted for letters in the Eriksen flanker task. Perhaps the most influential substitute has been replacing letters with arrows. Under ordinary circumstances, letter stimuli are not associated with left or right directional reactions. However, arrows are highly overlearned directional symbols that are widely used in society (traffic lights, directions on maps and streetways, etc.). The prototypical shape of an arrow is so ubiquitous and learned so early in life that it conveys spatial directional meaning automatically—even young children know immediately the direction indicated by an arrow. The introduction of arrows into the flanker task not only circumvented the extra time required to learn arbitrary letter-response mappings, but allowed for more straightforward tests of the contribution of response competition to the flanker effect by permitting the introduction of well-characterized experimental factors that act at the response end of processing (e.g., stimulus–response compatibility; i.e., the instruction to respond in the same or opposite direction of the target arrow).

In a study that was to have a major impact on the field at large by introducing the arrows version of the Eriksen flanker task (henceforth termed the arrows flanker task), Stoffels and van der Molen (1988) examined the effects of task-irrelevant noise on the visual choice reaction process. They combined the Eriksen flanker task with the (by now also classic) Simon task (Simon, 1982). In both tasks, task-irrelevant stimulus features (stimulus location in the Simon, flanker identity in the Eriksen) facilitate the designated response on some trials, but trigger competing response alternatives on others. In one experimental condition, key for present purposes, the flanker array consisted not of letters, as in the traditional Eriksen flanker task, but of arrows. The arrow arrays contained congruent (e.g., →→→→→), incongruent (e.g., ⟵⟵→⟵⟵), or orthogonal flankers (e.g., ↑↑⟵↑↑, ↓↓→↓↓). The visual arrow array was accompanied by a monaural (left or right ear) or binaural (both ears) tone for creating corresponding, noncorresponding, or neutral Simon effect trials.

**Fig. 1:**
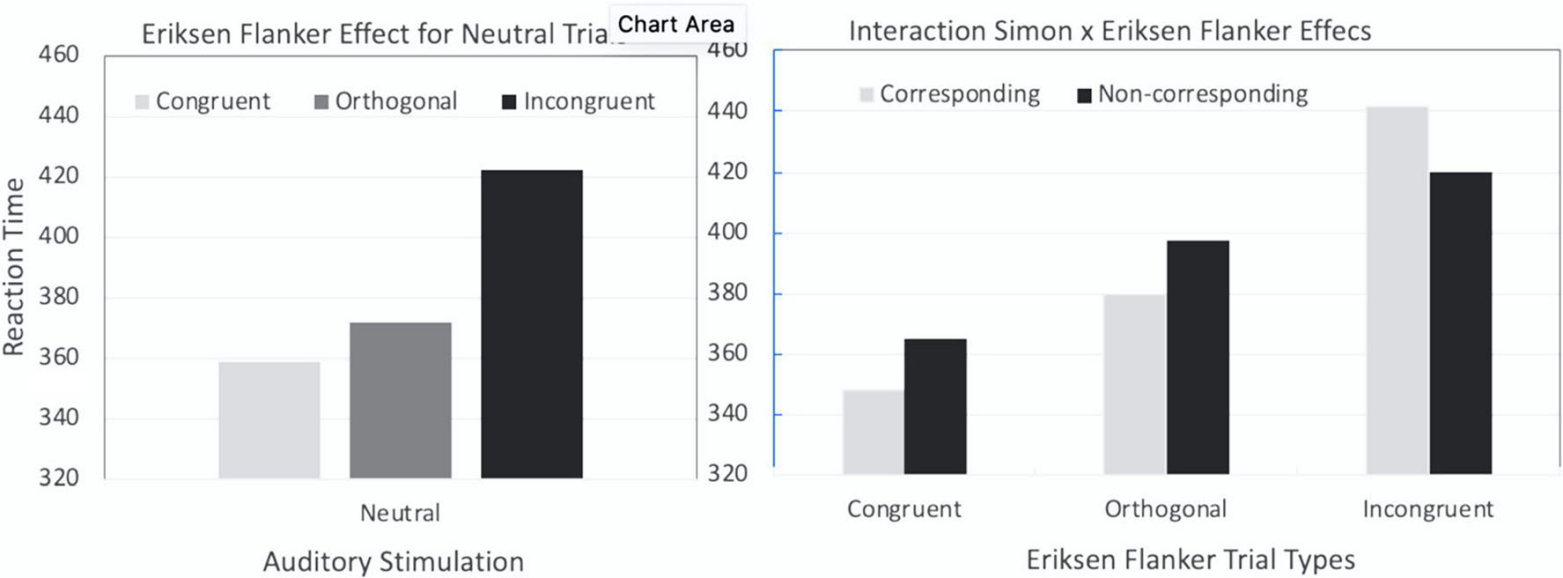
Left panel: Flanker effect on response speed on trials with binaural (neutral) stimulation. Right panel: Simon effect on response speed on trials with congruent, orthogonal and incongruent arrow flankers. (Redrawn from Stoffels & van der Molen, 1988)

With binaural stimulation, the results yielded the typical Eriksen flanker effect: Responses were fastest to congruent trials, slowest to incongruent trials, and in-between to orthogonal flanker arrays. With monaural stimulation, congruent and orthogonal flanker trials were associated with the typical Simon effect: Responses were fastest to corresponding trials, slowest to noncorresponding trials (and in-between to neutral, binaural tones; see Fig. 1, left panel). However, on incongruent flanker trials, the results showed a reversal of the Simon effect—that is, the speed of responding was slower on corresponding than on noncorresponding trials (see Fig. 1, right panel). Stoffels and van der Molen interpreted the observation of the typical Simon effect on congruent flanker trials and its reversal on incongruent flanker trials in terms of a crosstalk between the location information associated with the auditory stimulation on the one hand and the visual flanking arrows on the other. The idea is that location cues as generated by monaural stimulation and by arrow flankers, becoming available early during auditory and visual processing, interact. A conflict arises when cues are opposite and the resolution of this conflict will delay the required response (i.e., on congruent/noncorresponding and incongruent/corresponding trials).

Since the seminal publication of the arrows flanker task (Stoffels & van der Molen, 1988), there has been an abundant growth of studies using arrows as stimuli for the Eriksen flanker arrays. The arrows flanker task has been employed in hundreds of studies, and thus has become extraordinarily influential.

One notable example is provided by studies examining attentional networks using the Attention Network Task (ANT; Fan, McCandliss, Sommer, Raz, & Posner, 2002). Posner and Petersen (1990) had proposed that the anatomical brain areas supporting attention can be decomposed into the alerting, orienting, and executive networks. The alerting system is involved in maintaining a vigilant state, the orienting system serves to direct attention to a specific location in space, and the executive system is recruited when tasks require the resolution of conflict. The ANT paradigm, basically consisting of a cued arrows flanker task, was devised to assess these three networks. A central cue preceding the arrow array is supposed to activate the vigilance system; an eccentric cue activates the orienting system and directs the attentional system to a particular location; and the need to resolve conflict elicited by incongruent flankers is thought to recruit the executive system. Fan et al. (2002) observed that both alerting and directional cues facilitated the speed of responding, whereas incongruent compared with congruent flanking arrows delayed the speed of responding. Similar effects were reported in a subsequent study examining the brain regions involved in each of the networks (Fan, McCandliss, Fossella, Flombaum, & Posner, 2005), revealing that alerting, orienting, and flanker incongruence recruited dissociable networks in the brain. Subsequent work has indicated that executive action control entails two separate classes of processes: *Online* processes of action control can be distinguished from the *anticipatory* processes that modulate them (Braver, Gray, & Burgess, 2007; Davranche & McMorris, 2009).

## Arrow flankers and online action control: Response capture and selective suppression

Anticipatory and online control processes can be dissociated in terms of underlying neural networks, temporal dynamics, and sensitivity to experimental manipulations as well as individual differences. Online action control is exerted to suppress and overcome incorrect, inappropriate, or undesirable actions in favor of intention-driven action selection (Baumeister & Vohs, 2004), as, for instance, in overruling the habit of driving on the right side of the road when navigating traffic in Britain. Online action control in situations of multiple simultaneous action affordances entails at least the following component processes: first, prompting the activation of appropriate actions based on *intention-driven action selection*; second, resisting the activation of inappropriate actions based on extraneous stimulus–action associations that are strong enough to incur *response capture*; and third, suppressing the activation of inappropriate actions through active *response inhibition*. These will be discussed in more detail below.

Especially when appropriate actions compete for activation with strong alternatives, online action control may be needed to resist interference from these alternatives and ensure the timely and uninterrupted activation of the selected response (Miller & Cohen, 2001). Here we will return briefly to the previous discussion of continuous flow versus stages-of-processing conjectures, as these will take us to the currently predominant conceptualization in terms of dual-process models.

The capacity of the discrete stages-of-processing model to account for interference effects in the arrows flanker task was explored in detail by Ridderinkhof, van der Molen, and Bashore (1995). They were especially interested in determining if stage robustness, offered by Gopher and Sanders (1984) as a rigorous test for the stages-of-processing model, applied to the processing of both single-element and multi-element stimuli. Stage robustness asserts that the statistical relations between two factors should not change with the systematic addition of other experimental factors. Ridderinkhof et al. found that stage robustness did not generalize to the arrows flanker task. Specifically, in a series of experiments, they varied the perceptual salience (i.e., size) of the flankers relative to the target, and the symbolic compatibility of the response to the target. Response speed and accuracy are typically hampered by reduced stimulus discriminability (e.g., larger flankers) and by stimulus–response (S–R) incompatibility (e.g., a left-pointing target arrow designating a right-hand response). These factors had been demonstrated previously to produce strong additive effects (for review, see Sanders, 1990), suggesting discrete sequential stages of stimulus identification and S–R translation.

Ridderinkhof, van der Molen, and Bashore (1995) observed additive relations between variations in stimulus discriminability and S–R compatibility when target arrows were presented in isolation or flanked by response-neutral stimuli (♦, ↑, or ↓). However, these additive relations were transformed into overadditive relations when congruent as opposed to neutral flankers were presented, and into underadditive relations when incongruent flankers were presented, both of which are clear violations of stage robustness. The nature of the flankers determined whether variations in S–R compatibility were additive, overadditive, or underadditive with variations in discriminability. Most importantly, the cost of S–R incompatibility was reduced when incompatible responses were made to incongruent as compared with congruent arrays. This underadditive pattern could not be explained by either the discrete serial stages of processing or continuous flow model.

### Dual-process models

The underadditive pattern could be explained by reconceptualizing processing in the arrows flanker task within the context of dual-process models. A vast literature documents models that distinguish between *association-driven* and *intention-driven* processes (direct vs. deliberative, bottom-up vs. top-down, automatic vs. controlled, habitual vs. goal-directed, impulsive vs. deliberate, reflexive vs. reflective, involuntary vs. voluntary, and the like) and that seek to describe the respective contributions of those processes to behavior (Frank, Cohen, & Sanfey, 2009; Ridderinkhof, 2014). Although these models differ in details and domains, their common denominator entails an understanding of behavior in terms of the interplay between relatively automatic and relatively deliberative processes. Dual-process models have been entertained extensively in the field of action control (Kornblum, Hasbroucq, & Osman, 1990; Sanders, 1967), and have been embraced by many authors in the field. Basically, upon identification, a stimulus is thought to activate the correct response via a deliberate route, and to captivate activation of other (correct or incorrect) responses via a more direct processing route.

Back to the arrows flanker task. Ridderinkhof, van der Molen, and Bashore (1995) reasoned that target selection, target identification, and S–R translation occur over the deliberate route (akin to the discrete serial stages-of-processing conjecture). Processing of the full stimulus array takes place over the direct route (akin to the continuous flow conjecture), which bypasses the deliberate route. The S–R translation rule is implemented in the deliberate route, and hence cannot influence processing in the direct route. The output of the S–R translation process from the deliberate route (i.e., the response signaled by the target) is sent to the response activation level where it converges with response-relevant information sent via the direct route (i.e., the response signaled by the predominant flankers). At the response activation level, the response priming that has occurred via the direct route is integrated with the output from the deliberate route.

If the activated responses match, the motor program already activated via the direct route can be carried out quickly; but response competition ensues when the flankers signal a response that differs from the response associated with the target (i.e., both response alternatives have been activated). In case of mismatch, the motor program activated by the flankers must be aborted in favor of the alternative motor program, hence incurring a time cost. Thus, when compatible responses are required, congruent flankers prime the response signaled by the target, thereby facilitating the activation and production of the correct, compatible response. However, when incompatible responses are required, congruent flankers prime the alternative, incorrect response, thus producing response competition, which delays the production of the correct response. Similarly, when compatible responses are required to incongruent arrays, the flankers prime the alternative, incorrect response, whereas when incompatible responses are required to incongruent arrays, the flankers prime the correct, incompatible response, inducing no response competition and facilitating the production of the correct response. This predicted pattern provides an accurate fit to the underadditive findings reported by Ridderinkhof, van der Molen, and Bashore (1995).

### Timing is everything

Upon encountering stimuli that present multiple action affordances, as time progresses, action control processes will zero in on selecting the intention-guided action. Thus, during the early phase of processing, action selection is perhaps not yet perfectly intention guided, and hence is more vulnerable to potent action affordances, even if these are solicited by task-irrelevant flankers. As one well-documented result, responses that happen to be fast are more error-prone than those that happen to be slow: In the flanker task, incongruent flankers elicit many *fast* errors (Gratton, Coles, Sirevaag, Eriksen, & Donchin, 1988). Note, though, that these are gradual rather than all-or-none effects: During faster responses, action selection is driven *relatively* more by task-irrelevant affordances than by deliberate target-response translation; and on average, fast responses are *relatively* more error-prone for incongruent compared with congruent stimuli. This phenomenon is referred to as *response capture*. It is as if the action selection system is initially “hijacked” by the response activation as triggered by the flankers. Response capture is considered to be rapid, immediate, and nonreflective in nature.

Consistent with this typical pattern, the so-called activation-suppression hypothesis (Ridderinkhof, 2002a) asserts that processing of the information contained in the stimulus array is initially dominated by early activation of the response associated with the flankers, followed by the engagement and gradual buildup of deliberate processes, which in the case of conflict-inducing flankers produces selective suppression of the response information they convey. Beyond response capture, this hypothesis predicts that slow responses will have low error rates because the passage of time will have permitted the erroneous response information in the flankers to be suppressed so that the response is determined by the information contained in the target. These predictions have subsequently received ample and consistent empirical support (for review, see Ridderinkhof, Forstmann, Wylie, Burle, & van den Wildenberg, 2011).

### Action selection

Intention-based action selection is supported largely by two adjacent areas in the dorsomedial frontal cortex: the supplementary motor area (SMA) and the pre-SMA (Paus, 2001). The SMA has reciprocal connections with the primary motor cortex and with the spinal cord, whereas the pre-SMA is interconnected with other prefrontal areas rather than motor areas (Luppino, Matelli, Camarda, & Rizzolatti, 1993; Picard & Strick, 1996). These patterns suggest that the pre-SMA is involved in selecting and preparing actions, while the SMA is related to more downstream motor activation processes. Connectivity profile studies suggest a rostro–caudal continuum rather than a discrete division, with the rostral-most portions of SMA being more similar to adjacent caudal-most pre-SMA than to caudal-most SMA: In moving from pre-SMA to SMA, the functional significance of activation appears to shift gradually from being associated with cognitive aspects to being more tightly associated with motor aspects of action control (Picton et al., 2007). While SMA activation is seen only during action execution, the pre-SMA has been considered a key node for deliberate and voluntary action selection (Brass & Haggard, 2007).

**Fig. 2:**
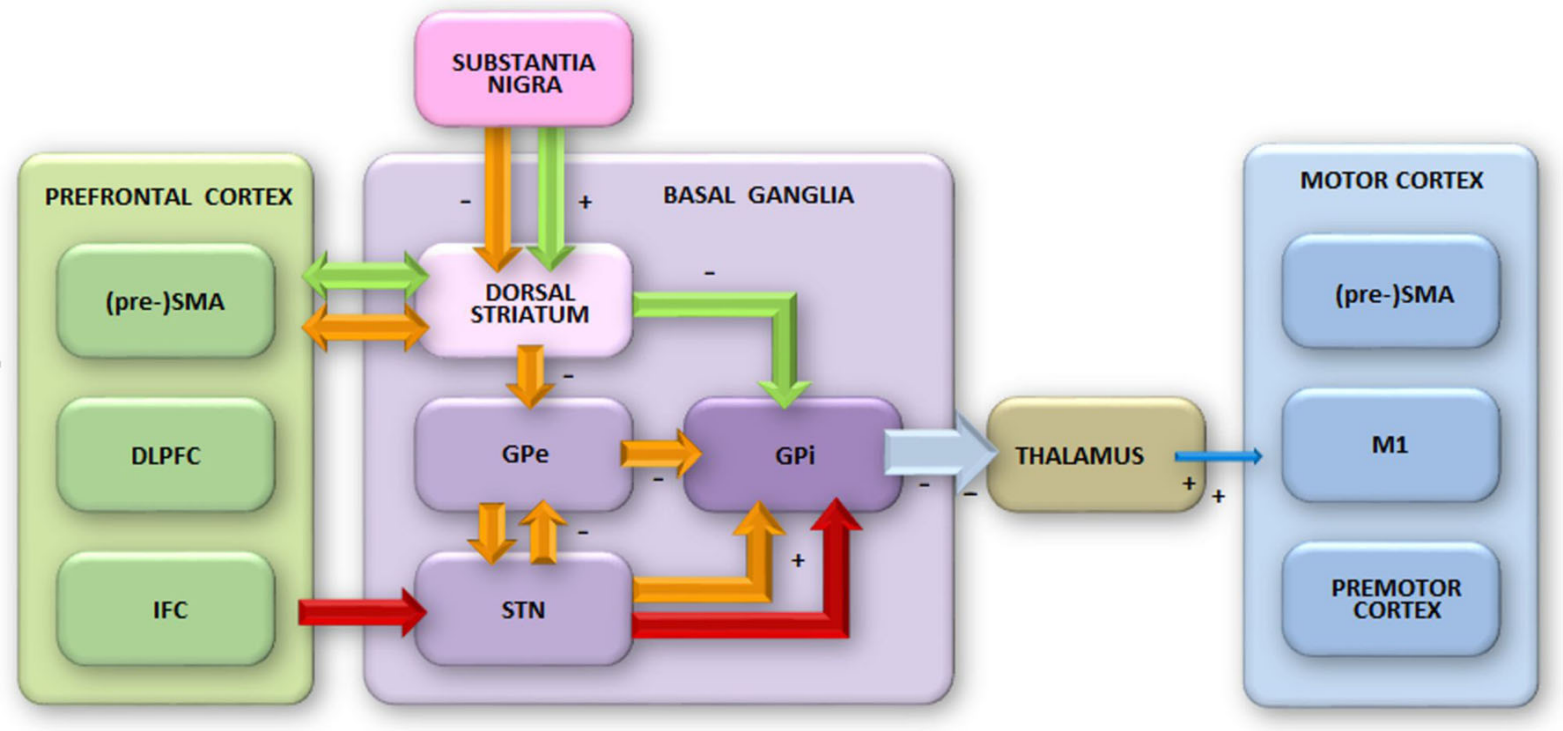
Schematic architecture of neural systems supporting action selection, response capture, and selective action suppression. The thalamus releases activation into the motor system, but is kept under tonic inhibition by the output of the basal ganglia until it is disinhibited by signals from the prefrontal cortex, that reach the thalamus via direct, indirect, or hyperdirect routes through the basal ganglia. Green arrows denote the direct route; orange arrows denote the indirect route; red arrows denote the hyperdirect route (see main text). SMA = supplementary motor area; DLPFC = dorsolateral prefrontal cortex; IFC = inferior frontal cortex; GPe = globus pallidus pars externa; GPi = globus pallidus pars interna; STN = subthalamic nucleus; M1 = primary motor cortex. (Color figure online)

The SMA and pre-SMA send efferents to the striatum and receive projections back from the globus pallidus pars interna (GPi) via the thalamus (Inase, Tokuno, Nambu, Akazawa, & Takada, 1999; see Fig. 2). In addition, the SMA and pre-SMA have hyperdirect projections to the subthalamic nucleus (STN; Isoda & Hikosaka, 2007); activations along these pathways presumably serve to keep basal ganglia output in check until voluntary action selection has completed (Bogacz, 2007).

### Response capture

Although Adam, like Eve, was curious to taste the fruit of the tree of knowledge, he had every intention to resist the temptation and to not eat from the apple. Likewise, in their pursuit of adequate task performance, participants in Eriksen flanker task experiments generally have the explicit instruction-based intention to select their actions based on the target and not on flankers. Yet the action affordances offered by these flankers are often difficult to resist, as if they capture the action system nondeliberately. Indeed, stimuli that present the individual with an action affordance (such as graspable objects) have been shown to activate the SMA even when there is no requirement to actually act on those stimuli (Grezes & Decety, 2002).

Often, action affordances like those triggered by extraneous stimuli are detrimental, as in the case of Eve holding the apple in Adam’s face. In the Eriksen flanker task, the flankers present task-irrelevant and often inappropriate action affordances. Intrinsic to the experience of such affordances is that stimuli incite or summon certain actions (Dreyfus & Kelly, 2007). However, the fact that stimuli may impel certain actions does not imply that execution of these actions is inevitable (Frijda, 2007): Temptations can be resisted, at least in principle.

The strength and the time course of response capture can be revealed by *conditional accuracy functions* (CAF) that plot accuracy rates as a function of reaction time (RT). In the Eriksen flanker task, fast responses are relatively more prone to errors than slower responses (Gratton, Coles, & Donchin, 1992). For a relatively large proportion of the fast responses, action selection is captured by flankers to such an extent that deliberate intention-driven action selection is bypassed and an overt response error is committed (for review, see Ridderinkhof et al., 2011).

When target and flankers compete for activation, selecting the appropriate action engages stronger activation of the pre-SMA compared with when response conflicts are absent (Ullsperger & Von Cramon, 2001). The strength of activation in pre-SMA co-varies with the extent to which inappropriate responses are captured by task-irrelevant stimulus features (Forstmann et al., 2008). Neurodisruption (Taylor, Nobre, & Rushworth, 2007) or lesions (Kennerley, Sakai, & Rushworth, 2004; Nachev, Wydell, O’Neill, Husain, & Kennard, 2007) of this region compromise the efficiency of action selection in the face of response capture by competing alternatives. The role of pre-SMA in the ability to select the appropriate response in the face of competing alternatives was confirmed further through a strong negative correlation between pre-SMA grey-matter volume and the susceptibility to response conflict (van Gaal, Scholte, Lamme, Fahrenfort, & Ridderinkhof, 2011). The amassed evidence points to a role for the pre-SMA as a gatekeeper that modulates the action-selection gate through which the available action affordances are translated into actual actions.

### Selective suppression

Inhibition is postulated as one of the mechanisms by which action control exerts its coordinating effects on subsidiary processes as implemented in other cortical and subcortical regions. Inhibitory control can be defined as the set of processes that results in the suppression of prepotent behavioral responses when such actions are premature or inappropriate in a given context and/or when such actions interfere with goal-directed behavior. According to the activation-suppression hypothesis, the rapid flanker-induced activation of an incorrect action is followed by the engagement and gradual build-up of online suppression of this affordance. Based on these temporal dynamics, slower reactions in conflict situations are less likely to be negatively impacted by incorrect action affordances because selective suppression has had more time to accrue. A host of studies now confirm that the interference from incorrect action affordances in conflict tasks levels off or reverses at the slow end of reaction time distributions, consistent with top-down suppression of the action affordance (for review, see van den Wildenberg et al., 2010).

One potential reason for interference leveling off, and even returning to zero at the slow end of the RT distribution, is decay of direct response activation of task (Hommel, 1994). This may indeed explain this distributional feature without having to resort to active suppression. However, a common finding for the Simon task, and a frequent finding for the arrow flankers task (although rarely observed for the Stroop task), is that the interference effect not only returns to zero, but reverses into a negative interference effect at the slow tail (for review, see van den Wildenberg et al., 2010). This pattern cannot be explained by passive decay, and is more readily reconciled with active inhibition (with selective suppression of the incorrect response yielding relative facilitation of the correct response). Departing from a variety of different assumptions, formal modeling efforts have begun attempts to address RT distributions in conflict tasks (e.g., Hübner, Steinhauser, & Lehle, 2010; Ulrich, Schröter, Leuthold, & Birngruber, 2015; White, Ratcliff, & Starns, 2011). However, the atypical feature of reversal of the conflict effect in slow responses thus far remains difficult to simulate; modeling efforts that entail selective suppression have not yet been published.

Of note, the magnitude of the reduction in the interference effect at the slow end of the RT distribution has been shown to be related to individual differences in the engagement of select prefrontal regions associated with inhibitory control (Forstmann et al., 2008; Forstmann et al., 2007). Based on classic monkey-lesion work, inhibitory control has been associated with the inferior frontal cortex (IFC; Iverson & Mishkin, 1970). In addition to the IFC, more recent studies point to a role for dorsolateral prefrontal cortex (dlPFC), pre-SMA, and several structures within the basal ganglia (see Fig. 2). The emerging patterns begin to delineate a picture in which the dlPFC is active in providing top-down guidance to action selection areas, the pre-SMA engages response inhibition as an instrument of action selection, the IFC is recruited to aid in implementing response inhibition in more demanding situations, and the basal ganglia keep all responses in check until the final signal is received from upstream.

The pre-SMA is optimally situated to transform the information coming in from association cortex into preparation for action—not only for action selection, as reviewed in preceding sections, but also for selective action suppression (Nachev, Kennard, & Husain, 2008; Picton et al., 2007). Areas within lateral PFC, including dlPFC and IFC, have been implicated in a large body of evidence as cardinal for response inhibition (for review, see Aron, Robbins, & Poldrack, 2014). Perhaps the most prominently reported frontal brain area involved in response inhibition in humans is the IFC, especially in the right hemisphere. Disruption of rIFC function through repetitive TMS has been reported to impair selective suppression of conflicting action tendencies (van Campen, Kunert, van den Wildenberg, & Ridderinkhof, 2018). In neuroimaging studies, comparison of incongruent to congruent trials revealed specific activations in IFC (e.g., Derrfuss, Brass, & von Cramon, 2004; Hazeltine, Bunge, Scanlon, & Gabrieli, 2003). Consistent with the notion that the IFC is engaged most prominently when strong inhibitory effort is required, strong correlations were observed between behavioral measures of selective suppression in conflicts tasks and both functional activation and structural connectivity in IFC (Forstmann et al., 2008; Forstmann et al., 2008).

Integration of the activation-suppression hypothesis into the dual-process model has generated a growing body of research that yields detailed characterizations of involuntary conflict effects in developmental and clinical populations. These studies, leaning heavily on the arrows flanker task, will be reviewed next.

## Arrow flankers and online action control in developmental and clinical populations

### Developmental trends in the arrow flanker effect

An early review of the literature on the development of selective attention suggested to Lane and Pearson (1982) “that more emphasis be given to understanding the basis of interference from irrelevant stimuli when it occurs. This focus, it is hoped, would facilitate the understanding of developmental changes in the degree to which irrelevant stimuli interfere with performance” (p. 317).

Studies using arrow flankers contributed considerably to a deeper understanding of developmental changes in the sensitivity to interference. An early study, conducted by Enns and Cameron (1987), presented three different age groups (4-year-olds, 7-year-olds, and young adults) with left-pointing or right-pointing arrows that were flanked by a single arrow pointing in the same (congruent trials) or opposite direction (incongruent trials). Their results showed that the congruence effect was significantly smaller for young adults than for the child groups, but, surprisingly, the effect obtained for 7-year-olds was larger than that for 4-year-olds. The authors interpreted their findings to suggest that children are more sensitive to flanker interference at the encoding stage of the visual processing of the target arrow, attributing the differences between child age groups to changes in speed–accuracy balance.

A child-friendly version of the ANT task (discussed above) was constructed, providing a convenient tool for assessing the development of the attentional systems (Rueda et al., 2004; for review, see Posner, Rothbart, & Voelker, 2016). In the child version of the ANT, arrow arrays were replaced by fish arrays; a central fish swimming to the left or right was flanked by four fish swimming in the same (congruent arrays) or opposite (incongruent arrays) direction. A direct comparison between the interference effects obtained with fish versus arrow flankers revealed that the effect was substantially smaller for fish than arrow arrays (see also Pozuelos, Paz-Alonso, Castillo, Fuentes, & Rueda, 2014; Simonds, Kieras, Rueda, & Rothbart, 2007). Several investigators thus continued to use arrow flankers (e.g., Johnson, Lewis, & Cornish, 2020; Lewis, Reeve, & Johnson, 2018; Mullane, Lawrence, Corkum, Klein, & McLaughlin, 2016), replicating the typical finding that congruence effects show a significant decrease with advancing age.

In broad outline, then, it seems fair to say that children suffer more from Arrows Flanker interference than adults. We can now begin to address Lane and Pearson’s (1982) question of how we should understand children’s susceptibility to interference elicited by irrelevant stimuli. One way to address this issue is by fractionating the visual reaction process to arrow arrays and then assess which segment of the reaction process is most sensitive to developmental change. In one study, Ridderinkhof and van der Molen (1995) used psychophysiological measures for segmenting the visual reaction process; and in a follow-up study applied additive factor logic (Ridderinkhof, van der Molen, Band, & Bashore, 1997).

**Fig. 3:**
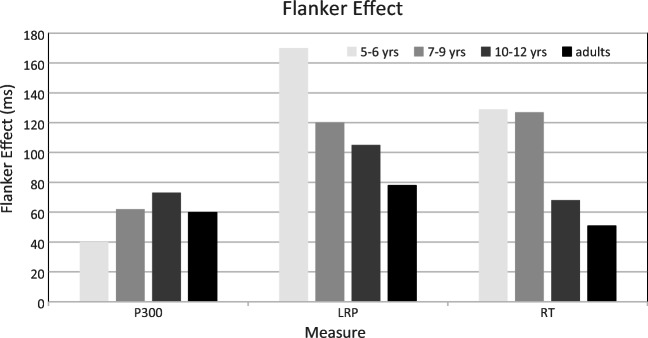
Developmental change in the arrow flanker effect (incongruent trials − congruent trials) on P300 latency (left panel), LRP onset latency (middle panel), and correct response latency (right panel) associated with the correct response. (Redrawn from Ridderinkhof & van der Molen, 1995)

Ridderinkhof and van der Molen (1995) used two brain potentials to decompose the reaction process into three major segments—that is, (a) the time-interval from the onset of the arrow array to the completion of stimulus evaluation, indexed by P300 latency; (b) the time interval between P300 latency and the onset of preferential response activation, indexed by the lateralized readiness potential (LRP); and (c) the time interval between LRP onset and the completion of the response executed by a left-hand or right-hand handgrip. As discussed above, in the adult literature, the study of the reaction process benefitted greatly from augmenting performance measures with psychophysiological indices of component processes (for review, see Coles; 1989; van der Molen, Bashore, Halliday, & Callaway, 1991). Participants in four different age groups (5–6-year-olds, 7–9-year-olds, 10–12-year-olds, and young adults) performed the arrows flanker task with a mixed presentation of congruent, incongruent, and orthogonal arrays. As anticipated, a substantial flanker effect on RT was observed, and this effect decreased with advancing age. The brain potential measures indicated that the developmental change in the flanker effect was manifested in the latency of LRP onset, but was not evident in P300 latency (see Fig. 3). The authors took this pattern of results to suggest that the locus of developmental change in the flanker effect is somewhere in between the identification of the target arrow and the cortical initiation of the correct response to this stimulus.

In their behavioral follow-up study in similar age groups, Ridderinkhof et al. (1997) manipulated processing at the encoding stage (by varying the size of the central arrow relative to the flanking arrows), at the response selection stage (by requiring participants to execute either a compatible or incompatible response to the central arrow (e.g., left-hand vs. right-hand response to a left-pointing arrow, respectively), and at the stage of response activation (by manipulating the time interval between a warning signal and the onset of the arrow array (a fixed vs. variable warning period). The results showed that congruent arrays attracted faster responses than incongruent arrays and, importantly, the flanker effect decreased with age. The combined results of congruity and compatibility were of most interest. On congruent trials, the speed of compatible responses was faster than incompatible responses, but on incongruent trials the typical compatibility effect reversed. The reversal of the compatibility effect on incongruent trials was interpreted to suggest that compatible responding is facilitated when congruent flankers prime the correct response, but delayed when incongruent flankers prime the incorrect response. In contrast, incompatible responding is facilitated when incongruent flankers prime the correct response but delayed when congruent flankers prime the correct response. Most importantly, S–R compatibility but not relative target size or warning period modulated the age trends in congruence effects. Along the lines of the dual-process conjecture outlined above, the authors explained the more pronounced flanker effect in children relative to adults by assuming that in children the processing along the deliberate route is less efficient, especially in the S–R translation stage, leaving more time for response priming by flankers. On compatible trials, incongruent flankers thus delay their response to a greater extent than in adults, but on incompatible trials, incongruent trials will facilitate the correct response more strongly.

The assumption that S–R translation is less efficient in children than adults is supported by a large body of research (for review see Cerella & Hale, 1994). Diffusion modelling, allowing a decomposition of the choice reaction process into the time needed for response selection and the remaining time associated with perceptual and motor processing, demonstrated that the bulk of the age differences in choice RT consists of the time needed for translating stimuli into responses (Ratcliff, Love, Thompson, & Opfer, 2012). Similarly, van de Laar, van den Wildenberg, van Boxtel, Huizenga, and van der Molen (2012) required compatible responses to single arrow stimuli and observed that the time interval between arrow onset and the onset of motor potentials over contralateral primary motor cortex was disproportionally larger in children than in adults. They observed that the dynamic balance of contralateral negativity associated with the activation of the correct response and ipsilateral positivity associated with the inhibition of the incorrect response that is present in adults was absent in children (see also Śmigasiewicz, Ambrosi, Blaye, & Burle, 2020). In addition, a larger proportion of partial errors (i.e., incorrect response activation beyond the corticospinal level) was seen in children than in adults. The findings support the notion that children are more susceptible to response conflict than adults.

This notion is supported further by the results of a large multisite study showing that developmental change in the sensitivity to the interference elicited by incongruent arrow flankers is associated with maturational changes in the anterior cingulate cortex (Fjell et al., 2012), a brain region widely believed to be involved in the detection and resolution of response conflict (for review, see Ridderinkhof, Ullsperger, Crone, & Nieuwenhuis, 2004).

In conclusion, the dual-process model proposed to account for developmental change in the sensitivity to flanker interference seems to provide a useful framework for examining the sources of arrows flanker interference effects in children. In what follows, we provide a comprehensive review of published studies illustrating how the arrows flanker task has been used to investigate interference control in clinical populations, focusing primarily on attention-deficit/hyperactivity disorders (ADHD) and Parkinson’s disease (PD).

### The arrows flanker task and the study of ADHD

Attention-deficit/hyperactivity disorder refers to a common neurodevelopmental disorder that is characterized by inattention, hyperactivity, and impulsivity (DSM-5; American Psychiatric Association, 2013). Emerging from the seminal paper “Stop, Look and Listen,” by Virginia Douglas (1972) was the notion that at the core of this childhood disorder, formerly known as “hyperactive/kinetic syndrome,” is an attentional deficit, a notion that is now inextricably linked to ADHD (cf. Mahone & Denckla, 2017). Both the clinical and experimental impact of the idea that children diagnosed with AD/HD suffer primarily from an attentional deficit were immediate and profound. It is not surprising, therefore, that after Douglas’ publication, investigators began to examine deficits in selective attention among children with this diagnosis using a wide variety of tasks, including the Stroop color-word task (for reviews, see Lansbergen, Kenemans, & van Engeland, 2007; van Mourik, Oosterlaan, & Sergeant, 2005;), the Simon and the Eriksen flanker tasks (for review, see Mullane, Corkum, Klein, & McLaughlin, 2009).

Despite the intuitive appeal of this idea and in contrast to the results from other selective attention tasks, such as the Stroop color-word task, studies using variants of the Eriksen flanker task have yielded mixed results. By and large, behavioral measures of mean processing speed have not reliably differentiated children, adolescents, or adults diagnosed with AD/HD from nonclinical control groups. However, suggestive patterns of increased error rates and diminished amplitude increases in the N2 component of the ERP among AD/HD-diagnosed groups, relative to controls, when challenged by incongruent flankers, have shown apparent deficits in response speed adjustments following commission of an error that may be linked to underactivation of neural systems thought to mediate response conflict inhibition, error recognition, and adaptive posterror motor adjustments. In this section, we provide a necessarily brief review of the extant literature and conclude that the failure of processing speed measures to differentiate groups consistently may reflect, in part, an underlying weakness in mean measures of response speed. We conclude this section by describing an example of how distributional analyses can dissect response processing speed with reasonable precision and expose group differences at a granular level that are not evident at the mean or molar level of analysis and, in so doing, suggest possibilities for establishing reasonably precise linkages between overt behavioral performance and underlying neural mediators.

In perhaps the first study examining the arrows flanker effect in AD/HD, Jonkman et al. (1999) compared the performances of children diagnosed with AD/HD and children with no clinical diagnosis using congruent and incongruent arrays, neutral arrays (a target arrow flanked by + signs), and unflanked single target arrows. They measured processing speed both behaviorally (response speed and accuracy) and psychophysiologically (P300 latency and amplitude). Overall, response speed was fastest to targets presented alone, intermediate to neutral and congruent arrays, and slowest to incongruent arrays; and P300 amplitude and latency were, respectively, higher and longer to incongruent arrays than to the other stimulus types. However, no group-related differences were found in response speed, P300 latency, or P300 amplitude to the various imperative stimuli. Only one difference was found: The size of the increase in error rate induced by incongruent relative to neutral arrays was larger among children diagnosed with AD/HD than controls. This pattern of results suggested to Jonkman et al. that the locus of the attentional deficit among children diagnosed with AD/HD, as reflected in flanker interference, is at the response not at the perceptual level of processing.

In contrast to Jonkman et al. (1999), however, evidence was found by Crone, Jennings, and van der Molen (2003) for processing deficits among children diagnosed with AD/HD at both early stimulus and late response levels of processing. Unlike Jonkman et al, Crone et al. included a stimulus that signaled withholding a response. Diagnosed and control children responded with the left hand to a left-pointing arrow, with the right hand to a right-pointing arrow, and made no response to a diamond-shaped stimulus. The target stimulus, shown at visual fixation, appeared alone, flanked by arrows, or flanked by the diamond-shaped stimuli. Stimulus types included single arrow, single-diamond, congruent arrow array, incongruent arrow array, central arrow flanked by diamonds, and central diamond flanked by arrows. Two comparisons are relevant here. First, responses were slower to congruent arrow arrays than to single arrows, and this slowing was more pronounced among children diagnosed with AD/HD than among control children. The suggestion—AD/HD compromises the child’s capacity to extract the central target from the flanking noise. Second, responses were fastest when flankers were congruent, slowest when they were incongruent, and in between when they were diamond shaped (i.e., signaled withholding a response). Once again, these differences were more pronounced for children diagnosed with AD/HD than for controls. The suggestion—AD/HD compromises the child’s capacity to suppress response competition induced by the flankers. These combined findings, unlike those of Jonkman et al. (1999), provided support for a double locus of the attentional deficit associated with AD/HD; an early filtering deficit and a later response control deficit. Like Crone et al. (2003), Konrad, Neufang, Hanisch, Fink, and Herpertz-Dahlmann (2006) and Johnson et al. (2008), using variants of the ANT, found that the responses of children diagnosed with AD/HD were slower, less accurate, and slowed to a greater extent by incongruence than those of control children.

However, Vaidya, Bunge, Dudukovic, and Zalecki (2005), using a variant of the arrows flanker task that was quite similar to the variant used by Crone et al. (2003) and included withholding a response to a designated stimulus, failed to find any response speed differences between children diagnosed with AD/HD and control children when responding to a central target flanked by congruent, incongruent, neutral (diamond-shaped), or no-go (*X*s signaling no response) stimuli. They did find, as did Jonkman et al. (1999), that error rate, not response speed, distinguished the two groups; the increase in error rate for incongruent relative to neutral arrays was larger among children diagnosed with AD/HD than among controls. FMRI analyses suggested that this differential increase in error rate may be associated with a differentially larger reduction of activation in neural pathways that mediate response inhibition (in particular, the caudate nucleus) compared with those that mediate interference suppression among children diagnosed with AD/HD (for related findings, see Liu et al. 2020; Plessen et al., 2016, discussed below).

It is quite apparent that these early studies yielded conflicting patterns of behavioral results that can muddle the interpretive landscape. A further example is found in Albrecht et al. (2008) who observed no performance differences between children diagnosed with AD/HD and control children on a version of the vertical arrows flanker task devised originally by Kopp, Rist, and Mattler (1996). They were the first to assess the moderating impact, if any, of AD/HD on the increase in amplitude of the N2 component of the ERP, recorded over frontocentral cortex, produced by flanker incongruence. In the variant of the task they used, the target arrow appeared at visual fixation after a brief delay following the appearance of two vertically aligned arrows, one immediately above and one immediately below fixation, pointing in either the same or the opposite direction of the target arrow. The increase in N2 amplitude invoked by incongruent arrays was smaller in children diagnosed with AD/HD than in control children. Previously, N2 amplitude had been reported to be sensitive to response incongruence but not to stimulus incongruence, suggesting that this ERP component is primarily sensitive to response conflict (Kopp et al., 1996; van Veen, Cohen, Botvinick, Stenger, & Carter, 2001). Accordingly, Albrecht et al. (2008) reasoned that AD/HD may compromise the child’s capacity to resolve response conflict.

The studies reviewed thus far present a heterogeneous pattern of results. Indeed, an early meta-analysis of Eriksen flanker studies examining the sensitivity to interference in AD/HD identified only seven relevant studies (Mullane et al., 2009). This report indicated that only two studies yielded the expected performance pattern (Crone et al., 2003; Konrad et al., 2006). More recent studies continue showing the same heterogeneous pattern. McLoughlin et al. (2009), for example, examined flanker effects in adults with AD/HD and healthy controls using a vertical variety of the arrows flanker task. More errors were committed on incongruent than congruent trials, but this effect was similar for both groups. The speed of responding was delayed on incongruent compared with congruent trials, but again, group differences were absent. Similar to the results obtained previously by Albrecht et al. (2008), N2 amplitude was more pronounced on incongruent than on congruent trials, and this effect was smaller in participants with AD/HD than in controls.

A comparable pattern was obtained by Wild-Wall, Oades, Schmidt-Wessels, Christiansen, and Falkenstein (2009), who used a vertical version of the arrows flanker task to assess adolescents with AD/HD. Congruent arrays were presented with 60% probability and incongruent arrays with 20% probability. On the remaining trials, a circle indicating that a response should be withheld replaced the central target arrow. Incongruent and no-go arrays induced more errors than congruent arrays and the speed of responding was delayed considerably on incongruent relative to congruent trials. This pattern did not differ, however, between groups. Extending the results obtained by Albrecht et al. (2008), Wild-Wall et al. observed that N2 amplitude was larger to incongruent and no-go arrays than to congruent arrays and that this increase was smaller in adolescents with AD/HD than in controls. A similar pattern was obtained by Johnstone, Watt, and Dimoska (2010), who tested children with AD/HD and typically developing children in an arrows flanker task with flankers that were either congruent, incongruent, neutral, or absent. More errors were made to incongruent than to neutral arrays, but error rates did not differ between neutral and congruent arrays. The speed of responding was also slower to incongruent than to neutral arrays. Again, the performance pattern did not discriminate between groups. Finally, in accord with the results of previous studies (Albrecht et al., 2008; McLoughlin et al., 2009; Wild-Wald et al., 2009), N2 amplitude was larger on incongruent than on congruent trials and the amplitude increase on incongruent trials was reduced significantly in children with AD/HD relative to controls. In a follow-up study, however, Johnstone and Galletta (2013) failed to observe a group difference in N2 amplitude to incongruent arrays and, similar to their previous studies, a group difference in the flanker effect on the speed of responding was absent.

More recently, Plessen et al. (2016) studied differences in brain regions associated with interference control and error processing between children with AD/HD and controls using a vertical arrows flanker task. They found, as had the bulk of the studies reviewed above, that the reduction in response speed and accuracy induced by incongruent arrays was comparable between the two groups. Most recently, Liu et al. (2020) examined the processing of arrow flankers in a relatively large sample of children and adolescents with AD/HD. Responses were slower and less accurate to incongruent than to congruent arrays, and the congruence effect on the speed of responding was, at trend-level significance, more pronounced in participants with AD/HD compared with controls. Consistent with previously reported results (Albrecht et al., 2008; Johnstone et al., 2010; McLoughlin et al., 2009; Wild-Wald et al., 2009), Liu et al. found an increase in N2 amplitude to incongruent relative to congruent arrays that was reduced in the participants with AD/HD.

Collectively, the data amassed to assess the alleged selective attention deficit in AD/HD using arrows flanker tasks paint a mixed picture. The majority of studies failed to uncover flanker effects on performance that reliably differentiated individuals diagnosed with AD/HD from nonclinical controls. The factors that distinguish those studies that did not find such differences from those that did have not been articulated with clarity, however. Several factors may have contributed to the heterogeneous pattern of results, including variations in diagnostic criteria, AD/HD subtype, comorbidity, and medication status (e.g., Mullane et al., 2009). Motivation may have also been an important contributor. It has been suggested, for example, that individuals with AD/HD are differentially sensitive to response contingencies (e.g., Crone et al., 2003; Douglas, 1999; Sergeant, 2000). In addition, variation in procedural details in how the task was structured and implemented could have contributed to the disparate pattern of findings (e.g., type of arrows flanker task, probability of incongruent arrays, time-interval between trials, the amount of practice to ensure reliable and robust RT patterns). The importance of procedural features is underlined by two meta-analytic studies examining interference in AD/HD using the Stroop task. When the difference between responses to the color-word card and the color card was taken as an index of the sensitivity to interference, the outcome of the meta-analysis failed to differentiate between individuals with versus without AD/HD (van Mourik et al., 2005). In contrast, a ratio score of sensitivity elicited in a Stroop task did yield a significant difference between groups (Lansbergen et al., 2007).

Before closing, one more study examining Arrows Flanker effects in AD/HD deserves consideration, as it entails more fine-grained analyses that suggest a direction that may be worthy of pursuing in this research domain. We close this section by presenting the case for doing so. Our case study is the reanalysis by Ridderinkhof, Scheres, Oosterlaan, and Sergeant (2005) of a subset of data from Scheres et al. (2003). Rather than limiting the analysis of performance to mean RT and error rate associated with each flanker condition, these authors used RT distribution analysis to assess group differences in the time course of interference control required when incongruent flankers elicit competing responses. The basic idea, outlined in previous sections, is that selective suppression requires some time to build up and become effective. Accordingly, flanker effects were found to level off or even gradually decrease with increasing response latencies. Guided by the hypothesis that conflict control is less efficient in individuals with AD/HD, it was anticipated that the decrease in the flanker effect associated with longer response latencies would occur later in individuals with AD/HD than in controls. Scheres et al. had observed the typical flanker effect on mean RT and accuracy; responses were slower and less accurate to incongruent arrays than to either congruent or neutral arrays, whereas the latter two arrays did not differ from each other. The flanker effect on the speed of responding was, at trend-level significance, more pronounced in children with AD/HD than in control children. Consistent with expectations, Ridderinkhof et al. observed that the flanker effect on RT levelled off for typically developing children whereas it continued to increase with longer response latencies for children with AD/HD. This pattern is consistent with the notion of inefficient response control in AD/HD, a notion that has received wide support from studies demonstrating that response inhibition is dysfunctional in AD/HD (for review, see Lipszyc & Schachar, 2010). In conclusion, this reanalysis underscored the potential value of RT-distributional analysis in studying the differential sensitivity of AD/HD to interference. The contribution of distributional analyses has also been demonstrated in other domains of AD/HD research (e.g., the use of ex-Gaussian parameters of RT (Galloway-Long & Huang-Pollock, 2018; Leth-Steensen, Elbaz, & Douglas, 2000), drift-diffusion parameters of the reaction process (Durston et al., 2010; Merkt et al., 2013; Metin et al., 2013; Shapiro & Huang-Pollock, 2019; Weigard & Huang-Pollock, 2017), and the analysis of respond and inhibit time distributions in AD/HD (Wiegard, Heathcote, Matzke, & Huang-Pollock, 2019).

### The arrows flanker task and the study of Parkinson’s disease

The arrows flanker task has also proven its usefulness in exposing deficits in cognitive control related to neurodegenerative diseases, such as in individuals diagnosed with Parkinson’s disease. The loss of dopaminergic producing neurons in the *substantia nigra pars compacta*, part of the basal ganglia (see Fig. 3), causes cardinal motor symptoms of the disease that include bradykinesia, rigidity, and tremor (Bjorklund & Dunnett, 2007; McAuley, 2003). As outlined in the previous sections, there is converging evidence from neuroimaging studies that supports the important role of frontal-basal ganglia circuitries in cognitive control to resolve conflict (cf. Casey et al., 2000; Forstmann et al., 2008; Zavala et al., 2013). The notion that the integrity of the frontal-basal circuitry is compromised in PD has inspired a broad range of studies that administered the Eriksen flanker task in order to investigate if and how cognitive control is affected in this clinical population.

Early studies by Praamstra and colleagues (Praamstra, Plat, Meyer, & Horstink, 1999; Praamstra, Stegeman, Cools, & Horstink, 1998) showed clear evidence that when compared with age-matched healthy controls, correct responses of individuals with PD are differentially slowed by the conflict produced on incongruent trials. The Praamstra studies showed tight linkages between patterns of exacerbated conflict and underlying EEG components, notably the LRP, the movement-related cortical measure discussed above. Analyses of the LRP indicated that the activation of the incorrect response on incongruent trials was enhanced in PD patients compared with controls. Additionally, cortical response-related activation started earlier in the clinical group, indicating that responding to visual signals in PD is associated with increased response capture that occurs earlier in time.

A clinical study by Wylie, Stout, and Bashore (2005) confirmed the behavioral pattern of exacerbated interference by incongruent flankers among PD patients reported by Praamstra et al. (1998), but extended it by testing whether the enhanced interference from incongruent distractors could be harnessed to the benefit of PD patients. Specifically, they had participants also make incompatible (opposite) reactions to the target arrow. On incompatible trials, incongruent flankers now signaled the correct response, whereas congruent flankers signaled the incorrect response. Both groups showed the typical reduction in the cost of incongruence on incompatible trials. However, the reduction was especially pronounced among PD patients. Thus, incongruent flankers could exacerbate response conflict or enhance facilitation among PD patients depending on the nature of the response decision. An interesting finding was that a particular subgroup of PD patients showing predominantly bradykinetic symptoms showed the most pronounced effects of incongruent flankers.

So, both Praamstra and colleagues (Praamstra et al., 1998), Praamstra et al., 1999 and Wylie et al. (2005) reported increased interference effects in PD compared with controls using the arrows flanker task. Alternatively, Lee, Wild, Hollnagel, and Grafman (1999) and Falkenstein, Willemssen, Hohnsbein, and Hielscher (2006) employed other variants of the Flanker task, a letter version and a vertical arrow version, respectively. Interestingly, they did not find significantly increased interference effects in their sample of PD patients when performance was compared with controls. In addition, the clinical sample sizes were quite small (i.e., *n* = 10 vs. *n* = 15, respectively). A color version of the flanker task also yielded comparable flanker effects between the clinical group (*n* = 20) and controls (Cagigas, Filoteo, Stricker, Rilling, & Friedrich, 2007). These mixed findings between various clinical studies might be caused by the marked design differences or by considerable interindividual variability within rather small PD subsamples.

This prompted Wylie et al. (2009a) to recruit a large sample of 50 medicated PD patients in order to replicate and extend their previous findings. Indeed, they replicated their behavioral pattern reported in 2005—namely, that PD patients show exacerbated costs of incongruence on the arrow flanker version. Furthermore, inspired by the activation-suppression hypotheses formulated by Ridderinkhof, 2002a, the authors compared flanker effects between groups as a function of reaction time. Response capture, or the susceptibility to making fast response errors to incongruent stimulus arrays, was comparable across groups. However, when compared with healthy controls, PD patients were less proficient in selective suppression of conflicting action tendencies for slower responses. Importantly, this marked deficit in selective suppression was only apparent in a subgroup of PD patients showing extreme costs of interference. Alternatively, about half of the patients showed behavioral patterns that were like the healthy control group. The magnitude of the flanker interference effect did not correlate with clinical features, such as disease duration, age of symptom onset, age, global mental status, and clinical motor rating scales. The clinical work described above confirms that cognitive control over responses, and selective suppression especially, depends on the integrity of fronto-basal ganglia loops.

The search for factors that modulate PD patients’ apparent vulnerability to response conflicts has provided important insights into PD. For example, Wylie et al. (2009b) tested how performance strategy might impact arrows flanker task performance among PD patients (*n* = 28) and controls. Performance strategy was manipulated by presenting different instructions to participants performing an arrow version of the flanker task. In the speed-pressure condition, instructions emphasized the importance to respond quickly, whereas in the accuracy-focus condition participants were instructed to make sure that they responded accurately. When focused on being accurate, PD patients where as efficient as healthy controls in resolving interference, as indicated by equivalent costs of incongruence. However, PD patients showed exacerbated costs of flanker incongruence when pressing for response speed that was related to less efficient selective suppression of conflicting action tendencies. Interestingly, even though the actual increase in response speed was marginal in PD, the mere perception of speed pressure was enough for disrupting action selection processes among some PD patients.

In an extensive study, Uc et al. (2014) followed a group of 43 participants diagnosed with PD to investigate the effects of extensive aerobic walking on motor function, quality of life and cognition. Those who completed the study walked three times a week for about 45 min each time for more than a year. Equipped with electronic heart rate and walking speed monitors, participants were enrolled in either an individual exercise condition or in a group. Half did interval training, and the other half did the continuous training, based on individual heart rate measures. Outcome measures after completing the walking program were compared with baseline measures collected before entering the program. The outcome measure taken from the arrows flanker task was the change in the percentage increase score (PIS) calculated using reaction times on congruent and incongruent trials: PIS = (((RT incongruent minus RT congruent) / RT congruent) × 100). When compared with their baseline performance, PD patients in the interval training group and those in the continuous group both showed significantly lower PIS scores, representing enhanced interference control as a result of exercise.

Surprisingly, studies of PD patients have universally been conducted while patients were taking their regular dopaminergic medication regime—for example, dopamine precursors or dopamine agonists. To our knowledge, there have been no studies to date that report the effects of dopaminergic medication on interference control using the arrows flanker task. However, the interference literature includes several within-subjects designs comparing PD patients while on dopaminergic medication versus off medication when performing on the Simon conflict task (e.g., van Wouwe et al., 2016; Wylie et al., 2012). Investigating how dopaminergic medications and deep brain stimulation therapies modulate incongruent flanker effects represents a potentially important direction for future studies.

As a concluding thought, we have used the arrows flanker task in our Parkinson’s disease and functional neurosurgery clinics (using our own PD and control norms) to identify patients who experience significant conflict effects so that we can educate them on strategies to mitigate their expression in real-world situations. For example, giving more space while driving so there is ample time to resolve conflicts, or navigating walking through crowds where conflicts in bumping into people can arise. Thus, the Eriksen flanker task has not only provided deeper insights into PD scientifically, but has aided and guided real-world decisions, precautions, and adaptations clinically.

## Arrow flankers and online action control in elite sports

The preceding section demonstrates how the arrows flanker task discloses, insightfully, patterns of deficits in the proficiency of cognitive control produced by various neurologic and psychiatric conditions, yielding converging insights into putative neural mechanisms underlying cognitive control and deepening our understanding of functional impact of disease. But what about cognitive control performance on the other end of the human spectrum; are there certain kinds of expertise and elite performance demands that might require superior cognitive control that could be unveiled by the arrows flanker task? This section outlines a relatively new research line in sports science that focuses of uncovering and quantifying exceptional cognitive control in professional athletes.

A wide range of fast-paced sports place considerable demands on the ability to make split-second reactions to a visual target stimulus (ball, puck, opponent player) that is surrounded by distracting stimuli. Perhaps individual skill in shielding reactions from the conflicting and interfering effects of incongruent flankers reflects a foundational and crucial skill for elite athletes to resist momentary response capture by distracting stimulus information and the responses it activates during play. In dynamic sports like baseball, soccer, hockey, and football, delays in resolving these processing conflicts on the order of a few tens of milliseconds (like those produced by incongruent flankers) can have significant ramifications for response execution speed and outcomes. Nearly 50 years after its inception, the Eriksen flanker task is making its mark in professional sports.

To our knowledge, two studies have investigated the hypothesis that elite athletes exhibit superior performance on the arrows flanker task. Using an arrow variant of the flanker task, Wang, Yang, Moreau, and Muggleton (2017) compared the performances of 18 elite athletes who played a visually and reactively dynamic sport (sometimes called *open* or *interceptive* sports; here, they studied elite athletes who played competitive badminton) and 18 athlete controls who competed in sports that are visually and reactively predictable and consistent (sometimes called *closed* sports; here, they studied elite athletes who ran track and field or competed in dragon-boat racing). The two groups of athletes performed with similar accuracy across flanker task conditions, but the badminton athletes outperformed the athlete control group in three aspects of performance: (1) their reaction times were significantly faster than athlete controls across all task conditions, (2) the variability of their reactions was significantly smaller than the athlete controls across all task conditions, and (3) the cost of incongruence on reaction speed was 20-ms smaller among badminton athletes than athlete controls. Thus, the elite badminton players were not only able to suppress interference more effectively, but they were able to do so while maintaining faster and more consistent reaction speeds.

The reactions of American football players are also executed in time-pressured, dynamic environments that are visually bombarded with distracting stimulus information. When a play begins, players move and react with exceptional speed and agility. Again, timing is everything. Delays or hesitations in reaction decisions (on the order of a few tens of milliseconds) produced by processing conflicts can have significant consequences, such as missed play opportunities, poorly timed actions, and blown assignments. In a series of investigations, Bashore and colleagues (Bashore et al., 2018; Wylie et al., 2019; Wylie et al., 2018) provided converging support to the hypothesis that highly talented, elite-level football players possess superior executive cognitive control skills. In the first of this series (Wylie et al., 2018), they compared 283 top National Collegiate Athletic Association (NCAA) Division I football players and nonathlete age controls on performance of the arrows flanker task. Consistent with prior work, the reactions of football players and controls were indistinguishable in terms of overall mean response speed and accuracy. However, a critical difference emerged. Football players showed a decisively smaller cost of flanker incongruence on RT (18% reduction) compared with controls. In fact, 20% of football players showed a cost of incongruence that was smaller than the best performing control participant. Thus, the reaction speeds of football players are less susceptible to response conflict from distracting information, suggesting more proficient cognitive control over response interference.

Additionally, differences in the magnitude of this interference control benefit were observed between specific football position groups. While the enhanced cognitive control was evident across both offensive and defensive position players, the defensive players showed significantly greater skill at controlling interference from incongruent flankers compared with the offensive players. Why might interference control be especially crucial for defensive players, and what clues might this differentiation offer to understanding the situations in which controlling interference from conflicting response tendencies is so important? The authors reasoned that a potentially insightful difference between offensive and defensive players involves strategic predictability. Offensive players execute predetermined plays where each player has a specific, individually assigned role before the play begins. Defensive players, on the other hand, have some general roles and assignments, but are unaware of what play the offense might run. Thus, defensive players are at a disadvantage in that there is unpredictability about how they may have to react. This uncertainty is routinely exploited by offenses who use visual misdirection, fakes, and illusiveness to create momentary conflict and confusion in a defender. Thus, in a visually dynamic and unpredictable environment, defensive players must be especially skilled at overcoming these conflicts produced by the offense in order to execute reactions to a desired target as proficiently as possible. These kinds of patterns and speculations provide considerable directions for future investigations.

From these initial studies, we see that the arrows flanker task unveils unique performance advantages among elite athletes. We anticipate considerable excitement and advancements in our understanding of these performance effects over the next decade. Some important questions to be answered concern the relative impact of experience and training on the development of these cognitive control skills in elite athletes, and how individual differences in flanker task performance predict in-game performance and contribute to predicting an athlete’s likelihood of performing successfully at higher levels of competition. To these ends, we are currently working with multiple teams across the National Football League and Major League Baseball to assess the predictive value of the arrows flanker task and other cognitive tasks to on-field performance, draft/recruiting selection, and customization of on-field training strategies that leverages the best science for modulating and improving these skills.

As a concluding thought, when the present first author was a young postdoc visiting Erik and Barbara Eriksen at their farm, more than 25 years ago, both of them were keen on learning the latest in the field, and were excited that their heritage (and in particular the Eriksen flanker task) was still so influential. However, both of them were (much) more excited about other issues, such as the sustainable ways of running their corn and soy farm, the holiday house they had built on their land, and, especially, the performance of their sons’ and grandsons’ football teams. They’d probably be thrilled to learn that the task that carries their name is now actually used to predict the role of cognitive control proficiency in high-level strategic skills and performance success in professional football and baseball.

We now turn to a final section in which we briefly discuss the role of the arrows flanker task in anticipatory action control.

## Arrow flankers and anticipatory action control: Posterror slowing and the congruence-sequential effect

Flexible, goal-directed adjustments of behavior require the continuous assessment of ongoing actions and the outcomes of these actions. The ability to monitor and compare ongoing actions and performance outcomes with internal goals and standards is critical for optimizing decision-making. What unfavorable outcomes, response errors, response conflict, decision uncertainty, and gut feelings have in common is that they signal that goals may not be achieved or rewards may not be obtained unless the level of cognitive control is subsequently increased. Although the literature on performance monitoring (for review, see Ridderinkhof et al., 2004) is beyond the scope of the present article, it is worth mentioning that the arrows flanker task has played a predominant role in that field as well (for review, see Ehlis, Herrmann, Bernhard, & Fallgatter, 2005; Huyser, Veltman, Wolters, de Haan, & Boer, 2011; Iannaccone et al., 2015). Importantly (for present purposes), evaluating the adequacy and success of performance is instrumental in signaling and implementing appropriate behavioral adjustments. For instance, detection of a performance error may be used to shift performance strategy to a more conservative speed–accuracy balance. The paradigmatic phenomena for studying such adaptive control have been posterror slowing and the congruence-sequential effect, to be discussed briefly below.

**Fig. 4:**
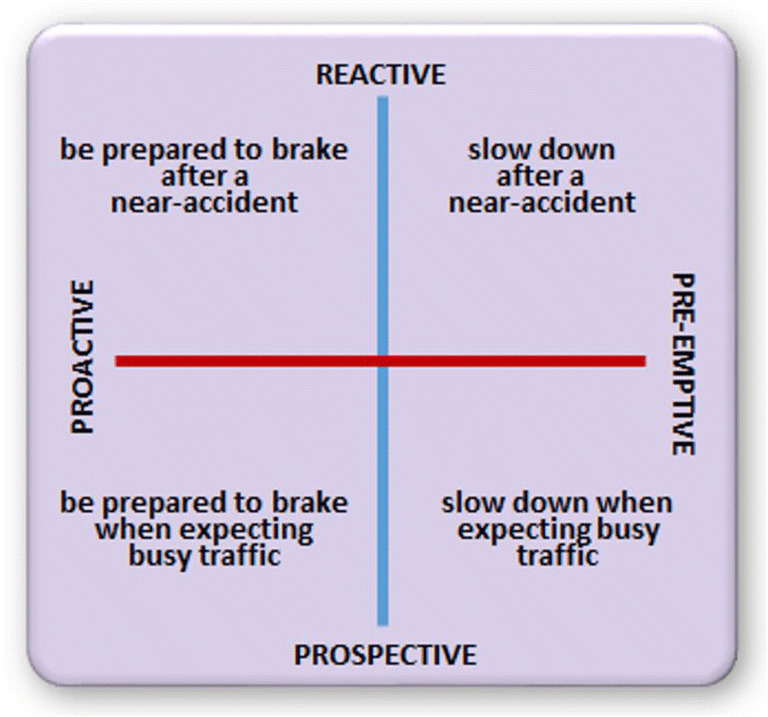
Anticipatory action regulation can be comprised of two dimensions:

A useful instrument in establishing action control is preparing for task-inappropriate action affordances and preparing to mitigate their undesired effects. Thus, the expression of the *online* action regulation processes may be modulated by *anticipatory* adjustments of action-selection priorities. Anticipatory action control refers to those modulatory processes that either strengthen online action regulation proactively, or preempt the need for such online control (Rabbitt, 1966). In participating in traffic, for instance, one’s responsiveness to action affordances is subject to fluctuations as a function of alarm signs, sudden crowding and/or slow-down of traffic, recent experiences (such as lane-drifting), the behavior of others, and so on—situations that one can try and prepare for. Such anticipatory processes can be described in terms of two orthogonal dimensions: regulation may be prospective or reactive in nature, and it may take on proactive or preemptive forms (see Fig. 4).

reactive versus prospective, and proactive versus preemptive

In many an instance, anticipatory regulation will be *reactive* in nature—that is, adjustments of online action control will be contingent upon performance errors or internal signals of performance difficulty, such as response conflicts. In other instances, anticipatory regulation will be more *prospective* in nature; for instance, one may slow down when anticipating busy traffic, or make use of explicit cues or instructions (e.g., traffic jams signaled by navigation software) to guide adjustments of processing priorities.

Irrespective of its prospective or reactive nature, anticipatory action control can be accomplished through either *proactive* or *preemptive* adjustments. One may attempt to proactively strengthen online action regulation, for instance, by a priori amplifying those processes that help keep our horses in check when a strong need for online regulation is anticipated. Alternatively, one may aim at preempting the need for online action regulation in the first place—for instance, by increasing the focus of attention to filter out flankers such that these fail to elicit the need for online control.

Perhaps the best documented varieties of *reactive* anticipatory adaptive control in the laboratory are posterror slowing (Rabbitt, 1966), posterror reduction of interference (Ridderinkhof, 2002b), and the congruence-sequential effect, often termed the Gratton effect after the first publication on the topic (Gratton et al., 1992). In the evolution of this congruence-sequential effect, the arrows flanker task has played an influential role.

### Once bitten, twice shy: The congruence-sequential effect

Of particular relevance is the discovery by Gratton et al. (1992) that the magnitude of flanker interference depends on the recent trial history. After facing conflict, people tend to adapt quickly to counteract possible detrimental effects of interference on subsequent occasions. In the Eriksen flanker task, these adaptive sequential effects are two-faced: (1) if the preceding trial was congruent, then the response on the present trial is faster than average if it is congruent again, but slower than average if it is now incongruent; and (2) if the preceding trial was incongruent, then the response on the present trial is faster than average if it is incongruent trial again, but slower than average if it is now congruent. Thus, adaptation depends on the level of conflict during the previous trial: If there was conflict, then control effort is increased (“closing the gates”); if there was no conflict, by contrast, then control can be relaxed (“opening the gates farther”).

Botvinick, Braver, Barch, Carter, and Cohen (2001) referred to this pattern as the *Gratton effect*, after its discoverer. Botvinick et al. offered the influential interpretation of the effect in terms of conflict adaptation. As a more theory-neutral term, Egner and colleagues (Egner, 2007; Egner, Ely, & Grinband, 2010) adopted the operational term *congruence-sequential effect* (CSE). Egner et al. demonstrated that the CSE steadily diminished with intertrial interval. Disappearing within 4 seconds, adaptive effects appear to be rather transient. This nontrivial finding argues against the original interpretation of the CSE proposed by Gratton et al. (1992), who emphasized the role of expectancy in preparing for future conflict. On this account, one would predict that the more time passes after a conflict trial, the better prepared one should be to handle further conflict, and hence CSE should increase. This prediction is clearly refuted by the Egner et al. findings.

Botvinick et al. (2001) attributed the CSE to attentional control, engaged to reduce the detrimental impact of task-irrelevant stimulus processing in the event of future instances of conflict. Such attentional control is strongest immediately following the eliciting conflict, but this resource-demanding attention dissipates as time elapses between trials. However, van den Wildenberg, Ridderinkhof, and Wylie (2012) noted that the CSE may pertain, at least in part, to *action* control rather than *attentional* control. Van den Wildenberg et al. review a series of studies (again prominently including the arrows flanker task) demonstrating that incongruent trials are followed by preemptively reduced capture as well as proactively augmented selective suppression of the response activated by flankers—these two phenomena of action control were discussed above.

## Psychometric properties of the arrows flanker task

The arrows flanker task as a powerful tool for clinical and diagnostic applications is bolstered by its solid test-reliability psychometrics in a number of studies covering a range of test intervals. Fan et al. (2002) have incorporated a variant of the arrows flanker task as a measure of executive control in their Attention Network Test (ANT). In 40 participants (mean age = 30; 77% females), they reported a same day test–retest Pearson reliability coefficient of .77 for the cost of incongruence. Similarly, Zelazo et al. (2014) tested the test reliability of the version of the arrows flanker task used in the NIH Toolbox (adapted from the ANT), reporting a cost of incongruence test–retest Pearson correlation coefficient of .85 and an intraclass correlation coefficient of .83 in 89 adults who completed the task after a mean interval of 16 days. Finally, Wöstmann et al. (2013) evaluated the test–retest reliability of the cost of incongruence in the arrows flanker task in 23 adults (mean age = 24 years, 70% females) twice, approximately 28 days apart, and reported a Pearson test–retest correlation of .69, an intraclass correlation coefficient of .91. MacLeod et al. (2010) used a permutation approach to estimate split-half reliability from a collection of 15 studies that used the ANT, reporting a weighted split-half coefficient of .66 and a Spearman–Brown reliability coefficient of .81 for the cost of incongruence. Note that although the costs of incongruence in Stroop, Simon, and Eriksen tasks are often considered to reflect similar underlying processes, many differences in terms of nonoverlapping component processes (e.g., semantic conflict, perceptual conflict), as well as differences in the degree of engagement of overlapping component processes (e.g., the need for attentional filtering and/or inhibitory control, the number of errors, the degree of posterror slowing), may contribute orthogonal variance to RT scores and hence suppress between-task correlations, as is commonly reported for “executive functions tests” (e.g., Stins, Polderman, Boomsma, & de Geus, 2005).

## Concluding comments

In the review presented in this article, we have attempted to describe the ontology of the arrows flanker task, to survey its applications over the 30 years since its inception, and to outline the impact it has made on the field of action control. Since the early studies with the Eriksen flanker task, focusing on the role of response competition in visual information processing, the arrows version of the task has over the years helped appreciate the processes of anticipatory and online action control involved in flanker interference effects in more detail. It has played a prominent and often central role in charting the developmental trends in action control, in describing and understanding individual differences in action control as related to neuropsychiatric disorders, and in unveiling the unique performance advantages owing to action control proficiency among elite athletes. It is our hope that by highlighting the impact of the arrows flanker task, this review will help honor the legacy of Erik Eriksen’s work.

## Footnotes

1 The Eriksens often used the terms *compatible* and *incompatible* rather than *congruent* and *incongruent*. The latter terminology became increasingly commonplace later on, especially in the arrows flanker task. In part, this was to distinguish the (in)compatibility between (the response designated by) the target and flanker stimuli from the (in)compatibility between the direction of the target arrow and the response it designated (with left-pointing arrows requiring a left-hand response in compatible S–R mappings but a right-hand response in incompatible mappings).
